# Factors Associated with Professional Mental Health Counseling Among Korean Adults: An Age-Stratified Analysis Using the Andersen Behavioral Model

**DOI:** 10.3390/healthcare14182946

**Published:** 2026-09-10

**Authors:** Jae Hee Kim

**Affiliations:** Department of Nursing, Daejin University, Pocheon-si 11159, Republic of Korea; hjw9266@daejin.ac.kr

**Keywords:** mental health services, professional counseling, Andersen Behavioral Model, loneliness, age-stratified analysis, Korea

## Abstract

**Background/Objectives**: Mental health service utilization remains considerably lower than the level of mental health need, and the factors related to utilization may differ across the life cycle. Applications of the Andersen Behavioral Model often underrepresent service availability and responsiveness. Based on the Andersen Behavioral Model, this study aimed to identify factors related to professional mental health counseling among Korean adults within three age strata. **Methods**: Using nationally representative, cross-sectional data from the 2021 National Mental Health Survey of Korea, this secondary analysis included 5511 participants: 1135 young adults aged 18–34 years, 3339 middle-aged adults aged 35–64 years, and 1037 older adults aged 65–79 years. Rao-Scott chi-square tests and survey-weighted logistic regression were used. The outcome was counseling for a mental health problem by a physician, nurse, clinical psychologist, or doctor of Korean medicine during the previous five years. **Results**: Female sex was associated with higher odds of counseling among young adults (odds ratio [OR] = 1.99, 95% confidence interval [CI] 1.01–3.95) and middle-aged adults (OR = 1.75, 95% CI 1.06–2.87), as was greater satisfaction with health status (OR = 2.67, 95% CI 1.46–4.91 and OR = 1.93, 95% CI 1.28–2.91, respectively). Higher loneliness was inversely associated with counseling in all three strata (OR = 0.70, 95% CI 0.55–0.89; OR = 0.69, 95% CI 0.58–0.83; OR = 0.64, 95% CI 0.52–0.80). **Conclusions**: Loneliness was the only factor associated with counseling in every stratum. These within-stratum estimates are not formal tests of between-group differences, and temporal order is uncertain. Loneliness may reflect a disruption in the pathway from need to help-seeking, change following prior care, or residual confounding; longitudinal evaluation is required.

## 1. Introduction

Mental health needs are high worldwide, while the response remains insufficient. The World Mental Health Report emphasized that mental health services need to be made more accessible and community-oriented [1]. In countries of the Organization for Economic Co-operation and Development (OECD), substantial treatment gaps persist [2]. In Korea, the 2021 National Mental Health Survey found that the lifetime prevalence of mental disorders among adults reached 27.8%, whereas only a minority of people with a recent disorder had received help from a mental health professional [3]. This demonstrates that a substantial gap exists between the need for and the actual utilization of mental health services.

Mental health service utilization reflects more than symptom burden. Prior research has identified sociodemographic characteristics, insurance and economic resources, perceived stigma, mental health literacy, social relationships, and the organization of local services as relevant factors [4,5,6]. The direction and magnitude of associations are not always consistent across populations. Greater clinical or psychosocial need may increase motivation to seek care, but the same circumstances can coexist with stigma, social withdrawal, low information access, financial strain, or limited service availability. In Korea, universal coverage through the national insurance system reduces some direct financial barriers, but concerns about structural discrimination, public prejudice, treatment records, adverse effects, and accessibility continue to shape psychiatric help-seeking [7].

The Andersen Behavioral Model has been widely used as a theoretical framework for explaining health service utilization across diverse populations [8,9,10,11]. It distinguishes predisposing characteristics, enabling resources, and need-related factors that may be associated with service use. This structure supports a multidimensional analysis rather than treating utilization as a direct function of symptoms alone. However, the model can be applied too narrowly when analyses focus almost exclusively on individual-level variables. Reviews and applications of Andersen-model studies have noted limited attention to contextual characteristics [12,13]. Complementary access frameworks, such as the patient-centred model proposed by Levesque and colleagues, place greater emphasis on service approachability, availability, affordability, and appropriateness [14]. The present dataset does not contain adequate indicators of service supply, waiting time, geographic access, responsiveness, continuity, or care quality. These omissions are treated as limitations rather than assumed to be represented by individual enabling variables.

Mental health need and service utilization may show different patterns across the life cycle. Young adults often report stigma, embarrassment, limited mental health literacy, and preferences for self-reliance as barriers to formal help-seeking [15]. Older adults may face negative beliefs about services, cost concerns, reduced mobility, and difficulty recognizing mental health problems [16,17]. Recent evidence also suggests that service use and expenditure have increased among young adults, particularly women, in some settings [18]. These studies indicate potential age-related heterogeneity, but they often focus on a single age group, a specific institution, or one class of determinants. In addition, separate age-stratified estimates do not by themselves establish statistically significant effect modification.

The present study contributes a nationally representative Korean analysis that organizes individual-level correlates of professional mental health counseling within the Andersen framework and presents estimates separately for three operational age strata. The strata were defined as 18–34 years, 35–64 years, and 65–79 years to represent early adulthood, middle adulthood and working-age years, and older adulthood within the survey age range, while retaining sufficient outcome events for exploratory regression. These cutoffs are analytical categories rather than universal developmental or clinical stages.

Using nationally representative data, this study aimed to identify factors related to professional mental health counseling among Korean adults based on the Andersen Behavioral Model and to estimate these associations within each age stratum. The study did not formally test every covariate-by-age interaction; therefore, comparisons across strata are descriptive and are interpreted cautiously.

## 2. Materials and Methods

### 2.1. Study Participants and Data

The study participants included 5511 respondents of the 2021 National Mental Health Survey of Korea. This study was a secondary analysis of this nationally representative, cross-sectional survey. The survey was conducted by the Ministry of Health and Welfare and the National Center for Mental Health from 19 June to 31 August 2021 [3]. The sampling frame covered the non-institutionalized population aged 18–79 years. Institutional residents and foreign nationals were excluded from the parent survey. Enumeration districts were selected using probability-proportional-to-size sampling, households were selected systematically, and one eligible adult was selected from each participating household. Of 13,530 sampled households, 5511 completed interviews, corresponding to a 40.7% response rate [3].

The survey used the Korean version of the Composite International Diagnostic Interview (K-CIDI), version 2.1, and comprised mental health indicators including mental disorders, mental health service utilization, perceptions of mental illness, quality of life, and social relationships. The present analysis included all 5511 survey respondents and applied the survey-provided stratification, cluster, and weight variables. No additional participant-level exclusion was applied before age stratification. Item nonresponse is reported in the descriptive table when available; regression analyses used observations with non-missing data for the variables included in each model.

In this study, participants were divided into three groups for analysis. The number of participants in each group was 1135 young adults (aged 18–34 years), 3339 middle-aged adults (aged 35–64 years), and 1037 older adults (aged 65–79 years). The inclusion of age 18 corrects the age range reported in the previous version and retains all survey-eligible respondents younger than 35 years.

### 2.2. Study Variables

**Figure 1:**
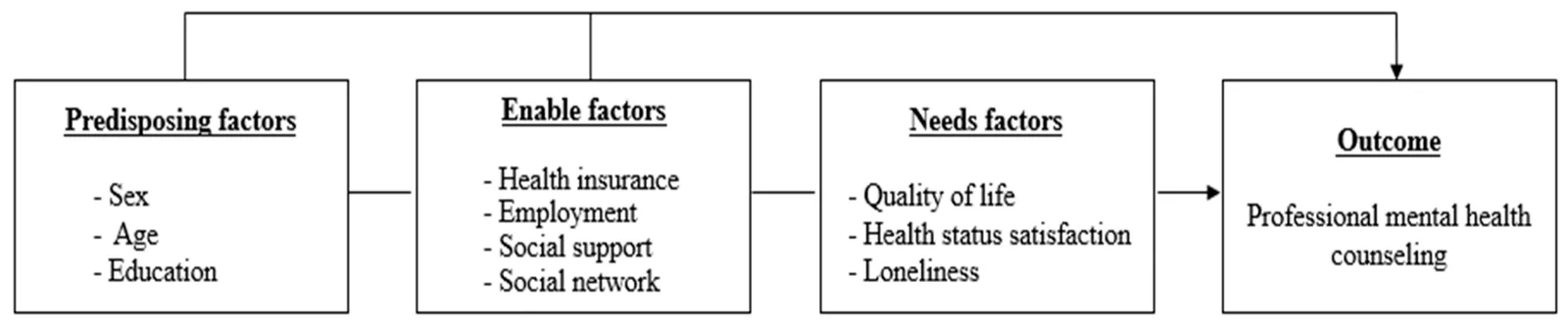
Professional mental health counseling based on the Andersen Behavioral Model.

Figure 1 presents the analytical organization of the variables. Predisposing, enabling, and need-related variables were treated as correlates of counseling rather than as temporally ordered causes. Contextual and service-system characteristics that were not available in the dataset are shown explicitly in the figure.

#### 2.2.1. Predisposing Factors

Predisposing factors are sociodemographic characteristics possessed before the need for mental health services arises; in this study, they comprised sex, age, and educational level. Age was categorized differently for each group: young adults were divided into 18–24, 25–29, and 30–34 years; middle-aged adults into 35–44, 45–54, and 55–64 years; and older adults into 65–69, 70–74, and 75–79 years. Educational level was also categorized differently for each group: high school or below versus college or above for young adults; middle school or below, high school, and college or above for middle-aged adults; and elementary school or below, middle/high school, and college or above for older adults.

#### 2.2.2. Enabling Factors

Enabling factors are socioeconomic characteristics that promote or impede mental health service utilization; in this study, they comprised health insurance status, employment status, social support, and social network. Health insurance status was classified into two categories, National Health Insurance and Medical Aid, and employment included both regular and non-regular employment. Because the Medical Aid subgroup was small in the young and older strata, estimates involving this variable were interpreted cautiously. Employment was available in the retained middle-aged and older-adult analyses; it was not included in the young-adult model, which limits direct comparability across strata.

Social support was measured using two items: “I can comfortably rely on friends or family.” and “There are people who can help me with everyday matters.” It was analyzed as a binary indicator and should be interpreted as a brief measure rather than a validated multi-item scale. Social network was measured using the item: “With how many people are you close enough to meet in person at least once a month, or to contact at least once a week, including family members, relatives, and friends?” The original analysis dichotomized the response as three or more people versus two or fewer people.

#### 2.2.3. Needs Factors

Quality of life, satisfaction with health status, and loneliness were classified as need-related factors for analytical organization. This classification does not imply that each variable is a direct or static measure of clinical need.

Quality of life was measured using a single item, “How would you rate your quality of life?”, with responses on a rating scale ranging from “very poor (1 point)” to “very good (5 points).” Satisfaction with health status was measured using a single item, “How satisfied are you with your health status?”, with responses on a rating scale ranging from “very dissatisfied (1 point)” to “very satisfied (5 points).” Single-item measures do not permit internal-consistency reliability estimation and may not capture the full multidimensionality of the constructs.

Loneliness was measured using two items (“I feel lonely.” and “I feel isolated.”) drawn from the instrument developed by Hwang et al. [19] to measure loneliness and social isolation. The two loneliness items were measured on a 4-point scale ranging from “almost never (0 points)” to “very much so (3 points),” with higher scores indicating greater loneliness. In this study, the Cronbach’s alpha values were 0.87 (young adults), 0.87 (middle-aged adults), and 0.88 (older adults).

#### 2.2.4. Outcome: Professional Mental Health Counseling

Mental health service utilization was defined as having received counseling from a professional (physician, nurse, clinical psychologist, or doctor of Korean medicine) for a mental health problem during the past five years. This item operationalizes a broad episode of professional counseling, not all possible mental health service utilization. It does not identify whether care was outpatient or inpatient, public or private, face-to-face or digital, or whether it involved psychotherapy, psychiatric consultation, emergency care, medication management, or repeated treatment. Medication obtained without counseling was not captured by this outcome. The five-year recall period was fixed by the parent survey and may increase recall error and obscure changes in service use over time [20].

### 2.3. Data Analysis Methods

Data were analyzed using IBM SPSS Statistics version 29. Because the National Mental Health Survey data are based on a complex sample design, this study used complex-sample analysis methods incorporating stratification variables, cluster variables, and survey weights. To examine differences in professional mental health counseling according to predisposing and enabling factors, the Rao-Scott chi-square test was performed. Logistic regression was conducted separately for each age group, with professional mental health counseling as the dependent variable.

All three need-related variables were entered in each regression because they were central to the study question. Predisposing and enabling variables were entered when they were associated with counseling in the Rao–Scott analyses. This screening strategy was used to limit model complexity given 62 counseling events among young adults, 138 among middle-aged adults, and 61 among older adults. The final models contained 5, 10, and 6 estimated predictor coefficients, excluding the intercept, respectively. The corresponding event-to-parameter ratios were approximately 12.4, 13.8, and 10.2. These ratios are only a rule-of-thumb assessment and do not eliminate the possibility of sparse-cell bias or unstable estimates [21].

Before logistic regression, the variance inflation factor (VIF) was calculated to check for multicollinearity. Generally, a VIF below 10 is considered to indicate the absence of a multicollinearity problem. In this study, the VIF for each age group ranged from 1.01 to 1.36 among young adults, 1.03 to 1.67 among middle-aged adults, and 1.02 to 1.97 among older adults. Odds ratios, 95% confidence intervals, and two-sided *p* values are reported. Statistical significance was defined as *p* < 0.05. No multiplicity adjustment was applied because the analyses were exploratory; therefore, isolated findings close to the significance threshold were interpreted cautiously. Formal age-by-covariate interaction tests were not conducted, so apparent differences in odds ratios across strata were not interpreted as statistically established between-group differences [22].

### 2.4. Ethical Considerations

The original National Mental Health Survey obtained informed consent from participants [3]. Although this study used data produced by the National Center for Mental Health, a governmental institution, it received an exemption from review from the Institutional Review Board (IRB) of Daejin University (exemption no. 1040656-202512-HR-02-20). Subsequently, a data-protection pledge was submitted to the National Center for Mental Health, and approval for data use was obtained.

## 3. Results

### 3.1. Differences in Counseling by Predisposing and Enabling Factors

Unweighted outcome counts were 62 users and 1073 non-users among young adults, 138 users and 3201 non-users among middle-aged adults, and 61 users and 976 non-users among older adults (Table 1A). Among young adults, the predisposing factor that showed a difference in professional mental health counseling was sex (*p* = 0.028), and the enabling factor was social network (*p* = 0.037). Counseling was higher among women than men and among those with a social network of two or fewer people than among those with three or more (Table 1B). The unweighted proportion reporting counseling was highest among older adults (5.9%), followed by young adults (5.5%) and middle-aged adults (4.1%). Employment was not included in the retained young-adult analysis and is therefore not shown in Table 1B.

Among middle-aged adults, counseling differed according to the predisposing factors of sex (*p* = 0.023) and educational level (*p* = 0.002). Counseling was higher among women than men and among those with middle school or below or high school education than among those with college education or above. Among enabling factors, differences were observed according to health insurance status (*p* < 0.001), employment status (*p* = 0.033), social support (*p* = 0.004), and social network (*p* < 0.001). Counseling was higher among those with Medical Aid than among those with National Health Insurance, among those without employment, among those without social support, and among those with a social network of two or fewer people. Among older adults, differences were observed according to sex (*p* = 0.006), health insurance status (*p* = 0.003), and social support (*p* = 0.003). Counseling was higher among women than men, among those with Medical Aid than among those with National Health Insurance, and among those without social support. These are unadjusted associations and should not be interpreted as independent effects.

### 3.2. Factors Associated with Professional Mental Health Counseling

To identify the factors associated with professional mental health counseling, logistic regression was performed for each age stratum (Table 2). In the adjusted young-adult model, female sex was associated with higher odds of counseling (OR = 1.99, 95% CI 1.01–3.95, *p* = 0.049), as was greater satisfaction with health status (OR = 2.67, 95% CI 1.46–4.91, *p* = 0.002). Higher loneliness was associated with lower odds of counseling (OR = 0.70, 95% CI 0.55–0.89, *p* = 0.004). The confidence interval for sex was close to the null and should be interpreted cautiously. Social-network size was not independently associated with counseling after adjustment (OR = 1.11, 95% CI 0.49–2.51, *p* = 0.802), and the direction of this estimate is opposite to the unadjusted association shown in Table 1; the estimate is imprecise and the change was not formally examined.

In the middle-aged model, female sex was associated with higher odds of counseling (OR = 1.75, 95% CI 1.06–2.87, *p* = 0.028). Among middle-aged adults, satisfaction with health status (*p* = 0.002) and loneliness (*p* < 0.001) were significantly associated with professional mental health counseling. Each one-point increase in satisfaction with health status was associated with 1.93 times higher odds of counseling, and each one-point increase in loneliness was associated with lower odds (OR = 0.69, 95% CI 0.58–0.83). Education, health insurance, employment, social support, and social-network size were not independently associated after adjustment, despite several unadjusted associations. The adjusted estimate for social support (OR = 1.36, 95% CI 0.80–2.32) is also directionally opposite to the corresponding unadjusted association and should not be interpreted as an independent effect.

Among older adults, only loneliness (*p* < 0.001) was significantly associated with counseling; each one-point increase in loneliness was associated with lower odds of counseling (OR = 0.64, 95% CI 0.52–0.80). The estimates for Medical Aid and several other variables were imprecise, with wide confidence intervals. Because no formal interaction tests were performed, the presence of a statistically significant association in one stratum and not another does not establish that the associations differ between strata. Employment was not associated with counseling in the bivariate analysis among older adults (*p* = 0.417) and was therefore not entered in the older-adult model.

## 4. Discussion

This secondary analysis identified several age-stratified associations with professional mental health counseling, but the results should be read as exploratory. Female sex was associated with higher adjusted odds among young and middle-aged adults, satisfaction with health status was positively associated among those two strata, and loneliness was inversely associated in all three strata. The older-adult model identified only loneliness at the conventional significance threshold and included imprecise estimates for some sparse categories. Because the models were fitted separately and no interaction tests were conducted, the study does not establish that the strength of associations differs by age.

The sex-related findings are consistent with literature suggesting that traditional masculinity norms emphasizing toughness, emotional control, and self-reliance may impede help-seeking among men [23,24,25,26]. However, these mechanisms were not measured in the survey. The estimates for female sex were modestly precise, and the lower confidence limit among young adults was close to 1. Accordingly, the findings support further examination of gender-sensitive access strategies rather than a definitive conclusion about causal mechanisms. Low-threshold entry points framed around sleep, stress, alcohol use, work functioning, or relationship difficulties may be more acceptable to some people than services framed solely as treatment for mental illness, but this policy interpretation requires direct evaluation.

The finding that higher loneliness was associated with lower professional mental health counseling suggests that the Andersen Behavioral Model should not be applied in the simplistic manner of “the greater the need, the greater the service utilization.” Loneliness may not be merely a variable reflecting the need for mental health services, but may indicate a point of disruption at which that need fails to translate into actual help-seeking behavior. Lonely individuals may have fewer opportunities to talk about their difficulties to those around them, a lower likelihood of obtaining service information, and a lack of people to recommend counseling or treatment [27,28,29,30]. In this sense, loneliness could operate as a relational barrier even when need is high. Nevertheless, the data do not establish this direction. Counseling may have occurred before the current loneliness assessment and may have reduced loneliness or related symptoms. Alternatively, current loneliness and prior service use may both reflect unmeasured diagnosis, symptom severity, functional impairment, socioeconomic resources, or service availability. Studies in other settings have also reported positive associations between loneliness, mental health need, and service use [31]. The present result therefore requires longitudinal replication with repeated measures before it can be interpreted as a barrier effect.

Among young and middle-aged adults, higher satisfaction with health status was associated with a higher likelihood of professional mental health counseling. This result differs from the usual interpretation of need factors. This should not be interpreted as evidence that better health causes service use. A favorable health evaluation may partly reflect health-management resources, confidence in navigating institutions, or willingness to engage in preventive care. It could also be a consequence of successful prior treatment, or it may capture constructs not distinguished by a single survey item. Research on single-item self-rated mental health suggests that global self-assessments can be related to functioning, service utilization, and broader health perceptions, but they do not map cleanly onto symptom severity [32,33]. Health literacy, self-efficacy, and stigma were not measured in the present study, so they remain hypotheses rather than demonstrated mediators.

Several enabling factors were associated with counseling in bivariate analyses but not after adjustment. This attenuation may reflect confounding or shared variance with other covariates, and in sparse subgroups it may also reflect limited statistical precision. The low variance inflation factors reduce concern about severe linear multicollinearity, but they do not rule out conceptual overlap or sparse-data bias. The very wide confidence interval for Medical Aid in the middle-aged model is a clear example of imprecision and should not be interpreted as evidence of either a large effect or no effect. In two instances the adjusted estimate was directionally opposite to the unadjusted association, for social-network size among young adults and for social support among middle-aged adults. Both adjusted estimates were imprecise, neither reversal was formally tested, and neither should be treated as a substantive finding.

The Korean context is relevant to policy interpretation. National insurance coverage does not eliminate stigma, perceived discrimination, concerns about medical records, treatment concerns, or local access barriers [7]. The present data support cautious consideration of age-appropriate and non-stigmatizing access routes, but they do not identify which specific program would be effective. For young adults, educational institutions, youth services, and confidential digital entry points may provide opportunities for screening and referral. A qualitative policy evaluation in Kunming emphasized coordination among schools, families, communities, and professionals, as well as unequal resource distribution and implementation challenges [34]. Because that study concerned Chinese middle-school students, it is a contextual example rather than direct evidence for Korean adults.

Digital environments may either facilitate or complicate help-seeking, and digital entry pathways to care should be distinguished from social media exposure and online coping. A cross-sectional study of high-school students in Kashmir reported associations between several forms of social media use and lower psychological well-being [35], and a systematic review found heterogeneous associations between social media use and depression, anxiety, or distress among adolescents [36]. Both concern adolescent populations rather than the present sample, and the National Mental Health Survey did not measure social media exposure, online coping, misinformation, or digital service use, so this study cannot determine whether digital engagement increased loneliness, substituted for face-to-face support, delayed formal care, or improved access. Digital entry pathways should therefore be evaluated for effectiveness, safety, privacy, and linkage to qualified care rather than assumed to be beneficial.

For older adults, the tentative finding for loneliness is compatible with approaches that integrate mental health identification into primary care, public health centers, home-visit services, senior welfare services, and community outreach. Korean research has linked social isolation and living arrangements with older adults’ mental well-being [37], while community-based outreach reviews suggest that active case-finding can improve contact with care in some settings [38]. The current analysis did not measure mobility, transportation, local provider supply, living arrangement, or referral pathways, so it cannot determine which linkage mechanism is most appropriate.

## 5. Strengths and Limitations

The strengths of this study are as follows. First, it analyzed factors related to professional mental health counseling among Korean adults using the nationally representative 2021 National Mental Health Survey data. Second, by applying the Andersen Behavioral Model to distinguish predisposing, enabling, and need-related factors, it structurally examined multidimensional correlates of counseling. Third, rather than analyzing all adults as a single group, it presented separate models for young, middle-aged, and older adults, making the distribution and uncertainty of associations across age strata visible.

However, several limitations exist. First, the cross-sectional survey measured current psychosocial characteristics but asked about counseling at any time during the previous five years. Recall bias is possible, and temporal ordering cannot be determined. A shorter recall period, such as 12 months, and repeated longitudinal assessments would improve interpretability. Second, the outcome does not distinguish frequency, duration, timing, adequacy, provider specialty, outpatient versus inpatient care, public versus private services, face-to-face versus digital care, psychotherapy, emergency services, or medication management. Medication-only treatment may have been missed.

Third, quality of life and health satisfaction were measured with single items, and the social-support variable was a brief binary indicator. The loneliness scale had acceptable internal consistency, but a two-item score cannot capture all relational or contextual dimensions. Fourth, important clinical and contextual variables were unavailable, including diagnosis, symptom severity, functional impairment, suicidality, stigma, mental health literacy, income, family structure, living arrangement, regional service density, travel time, waiting time, responsiveness, continuity, and care quality. Residual confounding is therefore likely.

Fifth, predisposing and enabling variables were screened using bivariate tests before regression, and the young-adult model did not include employment. This exploratory strategy may produce data-dependent selection and limits comparability. The models were not designed to provide formal effect-modification tests, and model-specific analytic sample sizes, need-related descriptive statistics by counseling status, standard errors, Wald statistics, and fit indices were not reported; these omissions reduce reproducibility and limit between-stratum interpretation. Sixth, the parent survey response rate was 40.7%, so nonresponse bias may remain despite weighting. Seventh, the survey was conducted in mid-2021, during the COVID-19 pandemic; service delivery and social interaction may not represent non-pandemic periods. Finally, sparse subgroups and wide confidence intervals limit precision, particularly for the Medical Aid and employment categories in the middle-aged model.

## 6. Conclusions

In this nationally representative secondary analysis, female sex was associated with higher odds of prior five-year professional mental health counseling among young and middle-aged adults. Among young and middle-aged adults, higher satisfaction with health status was associated with a higher likelihood of counseling. In particular, loneliness was associated with a lower likelihood of counseling among young, middle-aged, and older adults alike. These findings are associations and do not establish causal pathways or statistically significant differences between age groups. In particular, the temporal mismatch between current loneliness and retrospective service use means that loneliness may reflect a barrier to help-seeking, change after prior care, residual confounding, or more than one process. Policies that reduce stigma and provide coordinated, low-threshold routes to qualified care are reasonable areas for evaluation, but specific program effectiveness cannot be inferred from this study. However, because this study is based on a cross-sectional design, the direction and causal structure of the relationship between loneliness and counseling need to be further verified through future longitudinal studies. Future research should also use shorter recall periods, detailed treatment categories, clinical-need indicators, and contextual measures of service availability and responsiveness.

## Figures and Tables

**Table 1:** Professional mental health counseling by age stratum, predisposing factors, and enabling factors.

**Panel A. Unweighted Counseling Counts by Age Stratum**			
**Age Stratum**	**Total N**	**Counseling Yes, n (%)**	**Counseling No, n (%)**
Young adults, 18–34 years	1135	62 (5.5)	1073 (94.5)
Middle-aged adults, 35–64 years	3339	138 (4.1)	3201 (95.9)
Older adults, 65–79 years	1037	61 (5.9)	976 (94.1)
Overall, 18–79 years	5511	261 (4.7)	5250 (95.3)
**Variable**	**Category (n)**	**Counseling Yes, n (Weighted %)**	**Rao-Scott Chi-Square (*p*)**
**Panel B. Young Adults (N = 1135)**			
**Sex**	Male (568)	21 (4.1)	4.86 (0.028)
	Female (567)	41 (7.8)	
**Age (years)**	18–24 (385)	25 (7.4)	1.06 (0.346)
	25–29 (369)	19 (4.9)	
	30–34 (381)	18 (5.2)	
**Education**	High school or below (421)	27 (6.1)	0.05 (0.830)
	College or above (714)	35 (5.7)	
**Health insurance**	National Health Insurance (1075)	56 (5.7)	0.23 (0.634)
	Medical Aid (6)	0 (0.0)	
	No response (54)		
**Social support**	No (860)	46 (6.1)	0.17 (0.679)
	Yes (215)	16 (5.3)	
	No response (60)		
**Social network**	≥3 people (933)	43 (5.2)	4.38 (0.037)
	≤2 people (202)	19 (9.6)	
**Panel C. Middle-Aged Adults (N = 3339)**			
**Sex**	Male (1680)	55 (3.0)	5.22 (0.023)
	Female (1659)	83 (4.6)	
**Age (years)**	35–44 (999)	29 (2.6)	2.52 (0.082)
	45–54 (1149)	56 (4.1)	
	55–64 (1096)	52 (4.5)	
	No response (95)		
**Education**	Middle school or below (221)	17 (7.7)	6.46 (0.002)
	High school (1545)	70 (4.3)	
	College or above (1570)	51 (2.7)	
	No response (3)		
**Health insurance**	National Health Insurance (3262)	123 (3.5)	91.99 (<0.001)
	Medical Aid (54)	15 (30.0)	
	No response (23)		
**Employment**	Yes (2672)	88 (2.9)	4.57 (0.033)
	No (76)	5 (8.3)	
	No response (591)		
**Social support**	Yes (2372)	76 (3.1)	8.43 (0.004)
	No (967)	62 (5.3)	
**Social network**	≥3 people (2411)	68 (2.5)	31.39 (<0.001)
	≤2 people (928)	70 (7.1)	
**Panel D. Older Adults (N = 1037)**			
**Sex**	Male (509)	18 (3.5)	7.77 (0.006)
	Female (528)	43 (7.6)	
**Age (years)**	65–69 (574)	30 (4.9)	2.09 (0.125)
	70–74 (282)	23 (8.3)	
	75–79 (181)	8 (3.9)	
**Education**	Elementary school or below (287)	22 (7.6)	0.98 (0.375)
	Middle/high school (692)	37 (5.0)	
	College or above (58)	2 (5.0)	
**Health insurance**	National Health Insurance (981)	54 (5.3)	8.75 (0.003)
	Medical Aid (37)	6 (17.8)	
	No response (19)		
**Employment**	Yes (525)	18 (3.0)	0.66 (0.417)
	No (20)	1 (6.7)	
	No response (492)		
**Social support**	Yes (727)	33 (6.1)	8.74 (0.003)
	No (310)	28 (9.3)	
**Social network**	≥3 people (592)	35 (5.7)	0.01 (0.940)
	≤2 people (445)	26 (5.8)	

Notes: Panel A counts and percentages are unweighted and were calculated from the reported counts. Percentages in Panels B–D refer to the weighted population. No-response categories are displayed but were not treated as substantive categories in regression. ≥, greater than or equal to; ≤, less than or equal to.

**Table 2:** Factors associated with professional mental health counseling in age-stratified survey-weighted logistic regression.

Independent Variable (Reference)	Young Adults N = 1135; Events = 62 No Response = 0		Middle-Aged Adults N = 3339; Events = 138 No Response = 123		Older Adults N = 1037; Events = 61 No Response = 19	
	OR (95% CI)	*p*	OR (95% CI)	*p*	OR (95% CI)	*p*
Sex: female (ref. male)	1.99 (1.01–3.95)	0.049	1.75 (1.06–2.87)	0.028	1.72 (0.93–3.15)	0.082
Education: middle school or below (ref. college or above)	-		1.08 (0.43–2.73)	0.864	-	
Education: high school (ref. college or above)	-		0.96 (0.58–1.60)	0.880	-	
Health insurance: Medical Aid (ref. National Health Insurance)	-		3.01 (0.57–15.96)	0.195	1.89 (0.59–6.06)	0.286
Employment: no (ref. yes)	-		1.26 (0.32–4.97)	0.745	-	
Social support: yes (ref. no)	-		1.36 (0.80–2.32)	0.260	0.63 (0.32–1.23)	0.178
Social network: ≥3 people (ref. ≤2)	1.11 (0.49–2.51)	0.802	0.62 (0.35–1.11)	0.107	-	
Quality of life	1.29 (0.74–2.25)	0.372	1.18 (0.74–1.88)	0.496	1.14 (0.53–2.47)	0.732
Satisfaction with health status	2.67 (1.46–4.91)	0.002	1.93 (1.28–2.91)	0.002	1.21 (0.72–2.04)	0.475
Loneliness	0.70 (0.55–0.89)	0.004	0.69 (0.58–0.83)	<0.001	0.64 (0.52–0.80)	<0.001

Notes: OR, odds ratio; CI, confidence interval. Stratum totals and outcome events are shown. Model-specific analytic sample sizes may be smaller because of item nonresponse. Separate age-stratified models do not test statistical interactions between age stratum and each covariate.

## Data Availability

The restricted data are not redistributable by the author. Access is subject to the National Center for Mental Health application and data-use procedures.
